# Efficacy of Individualized Homeopathy in Treatment-Resistant Depression

**DOI:** 10.7759/cureus.18444

**Published:** 2021-10-02

**Authors:** Noheli Zepeda-Quiroz, Rodolfo Luna-Reséndiz, Jacqueline Soto-Sánchez

**Affiliations:** 1 Terapéutica Homeopática, Instituto Politécnico Nacional-Escuela Nacional de Medicina y Homeopatía (ENMH), Mexico City, MEX

**Keywords:** individualized treatment, homeopathy, arsenicum album, staphisagria, depression

## Abstract

Treatment-resistant major depression (TRD), defined as an insufficient response to at least two antidepressant treatments, is usually treated with antidepressants, psychotherapy, electroconvulsive therapy, repetitive transcranial magnetic stimulation, and vagus nerve stimulation or combinations of these. However, the response rate is modest and, on many occasions, insufficient or nonexistent. Here, we describe the case of a 19-year-old woman with a history of TRD, treated for depression since the age of five years. Her symptoms were resistant to fluoxetine, escitalopram, atomoxetine, and psychotherapy. Individualized homeopathic treatment with *Staphisagria*, *Nux vomica*, *Arsenicum album,* and *Lachesis trigonocephalus* was started. Posology was carried out in 200CH dynamizations. Treatment was prescribed for four months. This led to an immediate improvement in mood and a sustained and gradual reduction of depressive symptoms and, consequently, a reduction and then cessation of medication with antidepressants and psychotherapy. At follow-up eight months later, the patient is free of depression and medication. This case study reconfirms the usefulness of homeopathy in the treatment of depression. It also suggests that individualized homeopathic treatment may be useful in cases of treatment-resistant depression.

## Introduction

Major depression disorder (MDD) is one of the most prevalent neuropsychiatric disorders, affecting approximately 300 million people worldwide [1], characterized by impairments in cognition, memory, motor skills, motivation, emotional regulation, and the presence of neurovegetative symptoms. Patients with depression are more likely to develop chronic medical illnesses and drop out of treatment, to the detriment of their health [2]. Although the pathophysiology of MDD has not been fully understood, it involves several mechanisms, including serotonergic, noradrenergic, dopaminergic, and glutamatergic alterations, increased central and peripheral inflammation, hypothalamic-pituitary-adrenal (HPA) axis abnormalities, vascular changes, and decreased neurogenesis and neuroplasticity [2]. Current treatment focuses on antidepressants that inhibit monoamine neurotransmitter reuptake, elevate synaptic monoamine concentrations [3], and combine these with psychotherapy. However, less than 50% of patients respond to first-line antidepressant treatment or psychotherapy thus, in patients with treatment-resistant major depression (TRD), defined as an insufficient response to at least two antidepressant treatments [4], the use of electroconvulsive therapy (ECT), repetitive transcranial magnetic stimulation (rTMS), and vagus nerve stimulation (VNS) has also been employed with moderate efficacy. In recent years, probiotics as an adjuvant therapy have been proposed due to the relationship between the brain and the gastrointestinal system, with promising results [5]. Nevertheless, despite advances, many TRD patients do not respond to these therapies; in addition, most antidepressants have side effects, studies show that ECT produces adverse cognitive effects [6]. Therefore, the search for new, safer, and more effective therapeutic options for treating TRD demands comprehensive patient care. Homeopathic medicine takes into account the individuality of each patient for the selection of particular homeopathic medication. Several controlled and non-placebo controlled studies demonstrate that moderate to severe depression has been successfully treated with homeopathy, with efficacy superior to placebo and comparable to fluoxetine, and the safety of homeopathic treatment has also been demonstrated. In another cohort multiple randomized controlled trial study, the efficacy of homeopathic treatment in combination with the usual treatment alone was seen [7,8]. In the present study, we report a case of chronic depression successfully treated with centesimal potency homeopathy after an inadequate response to conventional therapies.

## Case presentation

A 19-year-old female patient, at five years of age she started psychotherapy for anxiety, anger, and depression; at six years of age, she was diagnosed with intellectual disability, with difficulty in writing, reading, and mathematics, for which she was prescribed treatment with fluoxetine and psychological therapy for three years, with a moderate response to the treatment. At 10 years of age, she suffered panic attacks, and at 16 years of age, she presented vasovagal syncope and bivalve aortic valve. Two years later, she was diagnosed with MDD and dysthymia and started treatment with different antidepressants (fluoxetine and escitalopram one-on-one in combination with atomoxetine at different doses) and intensive psychotherapy programs. All of them were ineffective. Due to the loss of control over her actions in recent months, the psychiatrist suggested that she be admitted to a psychiatric hospital. At her first homeopathic consultation, the diagnosis of MDD was confirmed according to the criteria established in the Diagnostic and Statistical Manual of Mental Disorders (DSM-5). On diagnostic evaluation, the patient scored 8/9 on DSM-5 criteria for MDD. According to what was evaluated in her previous clinical history, the patient presented treatment-resistant depression In homeopathy, each treatment requires careful individualization. Therefore, in the first consultation, the patient underwent a detailed homeopathic clinical history. The repertorization operation was performed with the computerized program Radar Opus version 10.0 (Zeus Soft, Isnes, Belgium). After repertorization (Table 1), homeopathic treatment with *Staphisagria* 200CH was started. Posology was carried out in 200CH dynamizations (five globules (Medicor) were dissolved in 250 mL of purified water and succussed 10 times before each dose). This posology was developed based on previous clinical experience where a mild, rapid and sustained response has been observed in treated patients. Follow-up consultations were performed at intervals of three to five weeks, as it is presented in Table 2. The selection of each medication was based on the totality of the patient's current symptoms at the time of consultation (Tables 3-5) until the patient's complete recovery was achieved. The homeopathic treatment was always accompanied by her psychotropic treatment (escitalopram and atomoxetine at different doses) and psychotherapy. Subsequent to the treatment with *Staphisagria *200CH the patient had an improvement in his general status, crying disappeared, thoughts of death, and insomnia began to decrease significantly, which allowed him to come out of isolation in her room. Aggressiveness, irritability, intolerance to contradiction were some of the symptoms that the patient manifested during her second visit, she was prescribed *Nux vomica *200CH, after five days she reported less irritability and increased tolerance with her relatives, especially with her younger sister, she did not get angry with her and they had fun together. She also apologized to her father for the first time, her surprised father hugged her. On her third appointment, her mood continued to improve as evidenced by the smile on her face and grooming. The patient reported fear of death, caused by the suicide of a friend. When the patient received *Arsenicum album* 200CH, her fear of death and mood improved markedly. After treatment with *Lachesis trigonocephalus *200CH, she reported very evident changes in her emotional well-being; she feels calm, shows patience in different situations with her nephews, is no longer jealous with her younger sister, and is loving with her parents, she resumes and starts new activities. After four months of treatment, she obtained a score of 1/9 in DSM-5, and her gastric symptoms such as colitis, gorgorism, stomach acid, and heartburn were alleviated. Periodic follow-ups performed every month until august 2021 revealed that the patient did not have any health problems related to depression, the patient is free of complaints, she has already finished a cosmetology course, and she has integrated to work as a model in a clothing store, and she is thinking of returning to a career in odontology. The patient's mother says she is very happy and surprised with the positive changes in her daughter since this has improved her quality of life. At the moment, she is no longer under psychiatric treatment. Informed consent was obtained from the patient for publication.

**Table 1:** Analysis and evaluation of symptoms for repertorization at the first consultation. List of abbreviations used: Staph - Staphisagria, Nat-m - Natrum muriaticum, Lac - Lachesis, Nux-v - Nux vomica, Ign - Ignatia amara, Ars - Arsenicum, Aur - Aurum, Lyc - Lycopodium clavatum, Calc - Calcarea (Radar Opus version 10.0).

	Staph	Nat-m	Lach	Nux-v	Ign	Ars	Aur	Lyc	Calc
	1	2	3	4	5	6	7	8	9
Total	13	11	11	11	10	10	10	10	10
Rubrics	28	27	24	22	23	22	22	20	19
Mind-ailments from-mortification	4	3	2	2	3	1	2	3	1
Mind-ailments from-love; disappointed	3	4	2	1	4	-	3	-	-
Mind-ailments from-anger	4	2	2	4	3	2	3	2	1
Mind-ailments from-anger-indignation; with	4	2	-	2	-	1	2	1	-
Mind-mental symptoms	1	2	3	2	3	3	3	3	2
Mind-envy	2	-	2	1	1	3	-	1	1
Mind-irresolution	1	2	3	2	3	2	1	2	2
Mind-hatred-revengeful; hatred	-	3	1	2	-	-	-	-	1
Mind-anxiety	1	2	2	2	2	4	3	3	3
Mind-mania-alternating with, depression	2	-	3	-	1	-	1	-	-
Mind-insecurity; mental	1	-	-	-	-	1	-	-	-
Mind-weeping-easily	1	2	-	-	-	-	-	1	2
Generals-obesity	1	3	1	1	1	2	2	2	3
Sleep-sleeplessness	3	2	3	3	2	3	2	2	3

 

**Table 2:** Homeopathic treatment prescribed to a patient with treatment-resistant depression.

Date	Symptoms	Homeopathic prescription
Sep 2, 2020	In the first consultation, the patient reported having ended a one-year relationship, expressing deep indignation about it, since her ex-boyfriend had another partner and was about to get married. She presented a slightly neglected physical appearance, weight gain, excessive physical and mental fatigue, deep sadness, involuntary crying at all hours, repressed anger and rage, feelings of worthlessness, lack of interest in everything, even in activities she used to enjoy doing, loss of concentration on specific or simple tasks, thoughts of death, loss of control over her actions and insomnia. She also expressed a desire to eat sweets and bread and said that her symptoms were worse at night and in hot places.	Staphisagria 200 CH (five globules in 250 mL of water, succussion) 10 mL every 12 h for 10 days then 5 mL every 12 h for three days.
Oct 2, 2020	The patient looks calm; the expression on her face is no longer deep sadness; however, she manifests aggressiveness towards her father, to whom she throws the phone, marked intolerance to contradiction, she admits feeling envy and jealousy towards her younger sister, she refers to having fears but does not know how to define them specifically, besides presenting an episode of tachycardia.	Nux vomica 200CH (five globules in 250 mL of water, succussion) 10 mL every 12 h for one week.
Oct 24, 2020	The patient presents with a better attitude, she looks happy, her grooming improves, and she is smiling when talking about the changes that are happening in her daily dealings with her family. However, she reports fear of death and anxiety since a friend of hers committed suicide a few days ago. She also expresses anger, hatred, and envy.	Arsenicum album 200CH (five globules in 250 mL of water, succession) 10 mL every twice a day for one week, followed by one dose daily for three days.
Nov 28, 2020	The patient attended her medical appointment, dressed up, and made up with a cheerful face, and starts a conversation about the activities she is having on the weekends since she has resumed practicing softball. There are no more traces of sadness; however, at similar intensities, some of the symptoms persisted: jealousy, envy, and hatred.	Lachesis trigonocephalus 200CH (five globules in 250 mL of water, succussion) 10 ml every twice a day for one week.
Jan 15, 2021	The symptoms of MDD are no longer present; there were no clinical signs of the disease. She no longer presents with jealousy, envy, and hatred. The patient is studying cosmetology and is working as a model in a clothing store.	No homeopathic medicine is prescribed
Mar 13, 2021	The patient has a high level of well-being and is very happy because her psychiatrist has decided to gradually withdraw the antidepressants.	No homeopathic medicine is prescribed

**Table 3:** Analysis and evaluation of symptoms for repertorization at the second consultation. List of abbreviations used: Nux-v - Nux vomica, Staph - Staphisagria, Tritic-vg - Triticum vulgare, Anac - Anacardium, Lyc - Lycopodium clavatum, Ars - Arsenicum, Ign - Ignatia amara, Puls - Pulsatilla, Hyos - Hyoscyamus.

	Nux-v	Staph	Tritic-vg	Anac	Lyc	Ars	Ign	Puls	Hyos
	1	2	3	4	5	6	7	8	9
TOTAL	7	7	7	7	6	6	6	6	6
Rubrics	14	10	10	9	15	14	14	14	12
Mind-despair	1	2	1	2	3	3	3	2	1
Mind-excitement-nervous-explosive	-	-	-	-	-	-	-	-	-
Mind-ailments from-jealousy	3	1	1	-	-	-	2	3	3
Mind-insecurity; mental	-	1	1	1	-	1	-	-	-
Mind-fear	2	1	3	1	3	3	3	2	2
Mind-fear-failure of	1	-	2	1	1	-	-	-	-
Mind-contradiction-intolerant of contradiction	3	2	-	2	4	1	3	1	1
Chest-palpitation; of heart	3	1	1	1	3	3	2	3	2
Mind-envy	1	2	1	1	1	3	1	3	3

**Table 4:** Analysis and evaluation of symptoms for repertorization at the third consultation. List of abbreviations used: Ars- Arsenicum, Calc- Calcarea, Tritic-vg- Triticum vulgare, Nat-m- Natrum muriaticum, Nux-v- Nux vomica, Lac- Lachesis, Anac-Anacardium, Sulph- Sulphur, Phos- Phosphorus.

	Ars	Calc	Tritic-vg	Nat-m	Nux-v	Lach	Anac	Sulph	Phos
	1	2	3	4	5	6	7	8	9
Total	7	7	7	6	6	6	6	6	6
Rubrics	18	15	9	14	14	13	12	12	11
Mind-anger	3	2	2	3	4	2	3	3	2
Mind-anxiety-future; about	1	3	2	2	2	2	2	2	3
Mind-sadness-alone-when	4	2	1	2	-	-	-	-	1
Mind-envy	3	1	1	-	1	2	1	1	-
Mind-hatred	1	2	1	4	2	2	3	3	1
Mind-discouraged	2	2	1	1	2	3	2	2	1
Mind-fear-death	4	3	1	2	3	2	1	1	3

**Table 5:** Analysis and evaluation of symptoms for repertorization at the fourth consultation. List of abbreviations used: Lac - Lachesis, Nux-v - Nux vomica, Ars - Arsenicum, Lyc - Lycopodium clavatum, Nat-m - Natrum muriaticum, Hyos - Hyoscyamus, Sulph - Sulphur, Calc - Calcarea, Staph - Staphisagria.

	Lach	Nux-v	Ars	Lyc	Nat-m	Hyos	Sulph	Calc	Staph
	1	2	3	4	5	6	7	8	9
Total	16	15	15	14	14	14	13	11	11
Rubrics	8	8	7	8	8	5	8	6	6
Mind-envy	2	1	3	1	-	3	1	1	2
Mind-jealousy	4	3	1	1	1	4	1	-	2
Mind-hatred-revengeful; hatred	1	2	-	-	3	-	-	1	-
Mind-despair	2	1	3	3	3	1	3	3	2
Mind-quarrelsome	2	3	2	2	2	4	3	1	2
Abdomen-inflammation-intestines	-	1	-	1	1	-	1	-	-
Generals-food and drinks-sweets-desire-headache, during	-	-	-	-	-	-	-	-	
Mind-malicious	2	3	3	2	1	2	1	2	2
Abdomen-inflammation-colon	1	1	1	1	1	-	1	-	-
Extremities-varices-lower limbs	2	-	2	3	2	-	2	3	1

## Discussion

Homeopathy is a therapeutic clinical medical method based on the application of the law of similars (like cures), morbid individuality (all human beings get sick in different ways, even if we have the same disease), single medication (each homeopathic medicine is unique and produces characteristic symptoms and clinical signs), and minimum dose [9], which allows the establishment of correct treatment, capable of achieving a gentle, effective and lasting cure. Here, we present a case of refractory depression, together with its respective homeopathic treatment, based on the previously mentioned principles. Since the age of 5, the patient had constantly resorted to antidepressants and psychotherapy, with continuous relapses. At the age of 18, she was about to be admitted to the psychiatric hospital. The aggravation in her depression seems to have been precipitated by the disappointment of her romantic love. Interestingly, the patient responded surprisingly well to treatment with* Staphisagria*, *N. vomica*, *A. album*, and *L. trigonocephalus* in parallel with allopathic treatment and had a good evolution; she presented significant improvements, which led to the decrease and cessation of the doses of conventional treatments and the total reestablishment of her depressive state. *Staphisagria* is a medicine used in homeopathy mainly for the treatment of nervous disorders aggravated by anger or repressed indignation. Being one of the main symptoms of the indignation presented by the patient after her love break-up, its use was preferred over *Natrum muriaticum*, which presents repressed anger to a greater degree. Studies in albino rats showed *Staphisagria* 30CH as an antidepressant [10]. *Staphisagria* contains flavonoids such as astragalin, which increase sleep rate and sleep time and decrease sleep latency in combination with sodium pentobarbital [11]. Rutin and isoquercitrin are active constituents of Staphisagria, with antidepressant activity [12,13]; rutin also decreases some symptoms of chronic stress such as anxiety and improves cognition and locomotor and muscle coordination skills. *N. vomica* acts mainly on the cerebrospinal nervous system. Our patient presented a marked tendency to violence and irritability. Therefore, she was prescribed *N. vomica*. In a study, *N. vomica* and *L. trigonocephalus* reduced depression, anxiety, and insomnia in climacteric women [14]. The administration of* N. vomica* 30CH improves the quality and quantity of sleep in individuals with a history of coffee-induced insomnia [15], it was also demonstrated that *N. vomica* (6CH, 12CH, and 30CH), significantly attenuates cognitive impairment and anxiety, in mice that exhibited its anxiolytic potential [16]. *N. vomica* contains strychnine, in this regard it was found that neurons in the lateral/basolateral amygdala express functional strychnine-sensitive glycine receptors, thus microinjection of strychnine directly into the basolateral amygdala reduces anxiety-like behavior in rats [17]. *A. album* is especially useful in patients who present fear of death or anxiety about the future, in this case, the patient showed these mental symptoms triggered by the suicide of her friend. Adler et al. reported the effective use of individualized homeopathic treatment, which included *A. album* in Q potencies for moderate to severe depression with results similar to those reported for fluoxetine [18]. Post-trauma anxiety was also effectively treated with *A. album *[19]. *L. trigonocephalus* is one of the drugs prescribed in patients showing jealousy with a tendency to aggressiveness; in this case, the patient presented intense jealousy from the beginning of treatment, which was not resolved with the treatment with *N. vomica.* However, treatment with *L. trigonocephalus* significantly improved this aspect and her emotional well-being. With respect to the time and benefits of the use of homeopathic treatment in this study, physical and emotional improvements were detected from five days of treatment, using 200CH doses, in accordance with other studies that used 200CH doses for the treatment of depression for six weeks [8]; although in these studies potentiation of the treatment was not applied as in the present study. It is also important to mention that the uses of 30C and Q potencies have been successfully employed for the treatment of depression [18] and their use depends on the characteristics of each case. This study has several limitations including sample size, lack of placebo, and follow-up period. However, it gives enough evidence for future well-designed randomized controlled studies to further evaluate the efficacy, effectiveness, and safety of homeopathic treatment alone or in combination with conventional medicine on patients with TRD, separating them from patients with other types of depression.

## Conclusions

TRD is a neuropsychiatric condition characterized by marked functional impairment in the individual due to its chronic, pharmacological options are limited and with moderate or no therapeutic effects for people with TRD. Here, we present the case of a 19-year-old woman diagnosed with TRD who was successfully treated with individualized homeopathy. This therapeutic approach resolved the depressive symptoms after a four-month treatment period and relieved the patient's gastric symptoms. Conventional psychiatric treatment was also completely suspended. Our case report showed sustained remission for eight months with no adverse effects or relapses has occurred so far. This case study suggests that individualized homeopathic treatment may be a useful complementary approach in treatment-resistant depression.
